# Hydrophobin Fusion of an Influenza Virus Hemagglutinin Allows High Transient Expression in *Nicotiana benthamiana*, Easy Purification and Immune Response with Neutralizing Activity

**DOI:** 10.1371/journal.pone.0115944

**Published:** 2014-12-26

**Authors:** Nicolas Jacquet, Catherine Navarre, Daniel Desmecht, Marc Boutry

**Affiliations:** 1 Institute of Life Sciences, University of Louvain, Louvain-la-Neuve, Belgium; 2 Department of Pathology, Faculty of Veterinary Medicine, University of Liège, Liège, Belgium; Icahn School of Medicine at Mount Sinai, United States of America

## Abstract

The expression of recombinant hemagglutinin in plants is a promising alternative to the current egg-based production system for the influenza vaccines. Protein-stabilizing fusion partners have been developed to overcome the low production yields and the high downstream process costs associated with the plant expression system. In this context, we tested the fusion of hydrophobin I to the hemagglutinin ectodomain of the influenza A (H1N1)pdm09 virus controlled by the hybrid En_2_PMA4 transcriptional promoter to rapidly produce high levels of recombinant antigen by transient expression in agro-infiltrated *Nicotiana benthamiana* leaves. The fusion increased the expression level by a factor of ∼2.5 compared to the unfused protein allowing a high accumulation level of 8.6% of the total soluble proteins. Hemagglutinin was located in ER-derived protein bodies and was successfully purified by combining an aqueous-two phase partition system and a salting out step. Hydrophobin interactions allowed the formation of high molecular weight hemagglutinin structures, while unfused proteins were produced as monomers. Purified protein was shown to be biologically active and to induce neutralizing antibodies after mice immunization. Hydrophobin fusion to influenza hemagglutinin might therefore be a promising approach for rapid, easy, and low cost production of seasonal or pandemic influenza vaccines in plants.

## Introduction

Influenza infections are of major concern for public health. Pandemics have caused more than 50 million deaths and cumulatively more have been caused by seasonal infections [1]. Vaccination has been widely used since the early 1960's to prevent contamination. Millions of doses are being produced yearly based on the well established egg-based system developed in 1941 [2]. Nevertheless, emerging problems have pointed out the need for new platforms for influenza vaccine production [3]. These alternative systems should be flexible regarding strain changes and rapid with respect to pandemics. Plants were recently proposed as a platform for vaccine production with the benefits of low cultivation costs, rapid biomass availability, ease of scaling-up, and limited risks of pathogen contamination [4]. Moreover, transient expression in leaves allows for the rapid and high-level production of pharmacological proteins [5].

Hemagglutinin (HA), the most immunogenic protein of the influenza virus, is the main target for recombinant influenza vaccine development. In plants, Influenza antigens were first expressed transiently in *Nicotiana benthamiana* leaves as a chimeric protein composed of an HA fragment fused to a fragment of neuraminidase, both from an H5N1 influenza strain, as well as to a thermostable lichenase [6]. Two approaches based on the expression of the HA protein alone were investigated. Firstly, full-length HAs from H1N1 (A/New Caledonia/20/99) and H5N1 (A/Indonesia/05/05) viruses were expressed transiently in *N. benthamiana* as virus-like particles (VLP) that bud from the plasma membrane [7]. A successful phase II clinical trial was achieved by Medicago with HA-VLPs from an avian H5 influenza strain [8]. Secondly, expression of a soluble truncated HA construct from A/Wyoming/03/03 (H3N2) was achieved by removal of the transmembrane domain and the addition of a KDEL retention signal [9]. This approach was used to express the HA from three 2008–2009 seasonal strains as well as the 2009 swine pandemic H1N1 (A/California/04/09) strain, an avian H5N1 (A/Indonesia/05/05) strain [10]–[11], and a low pathogenic avian H7N7 strain [12]. Recently, the soluble truncated HA from the pandemic A/California/04/09 (H1N1) was shown to be safe and immunogenic in a phase I clinical trial [13]. Both the VLP and the truncated HA approaches were shown to be a feasible response strategy to pandemics in developing countries, by the stable and transient expression of full-length or truncated HA from an avian H5 influenza strain in *Nicotiana tabacum* plants [14].

To enhance the accumulation level and to simplify the downstream purification procedure, the recombinant protein can be fused to a protein-stabilizing partner such as zein from plants, elastin from animals, and hydrophobin from fungi (reviewed in [15]). Hydrophobin I (HFBI), a small (∼10 kDa) surface-active protein secreted by filamentous fungi, possesses the ability to alter the hydrophobicity of the fusion partner, which can therefore be purified by an aqueous two-phase system (ATPS) [16]. This approach has been successfully used in *N. benthamiana* agro-infiltrated leaves and *N. tabacum* BY-2 cells for the expression of GFP in ER-derived protein bodies (PB) [17]–[18]. However, fusion of HFBI to the HA ectodomain from the Influenza A/Hatay/2004 (H5N1) virus in transgenic *N. tabacum* plants did not improve its expression compared with non-fused HA [19], while the fusion of elastin-like polypeptide (ELP) to the same HA ectodomain increased its accumulation level by 10-fold without compromising its functionality [19]–[20].

In the present study, we investigated hydrophobin fusion as a tool to obtain high-level expression of the recombinant HA ectodomain from the Influenza A/Texas/05/2009 (H1N1) virus by transient expression in *N. benthamiana* leaves. High expression levels of H1-HFBI in ER-derived protein bodies were obtained. H1-HFBI was easily and efficiently purified by ATPS. The immunogenic properties and the potency to induce neutralizing antibodies of the purified antigen were demonstrated by immunological studies in mice.

## Results

### Transient expression of H1-HFBI

**Figure 1:**
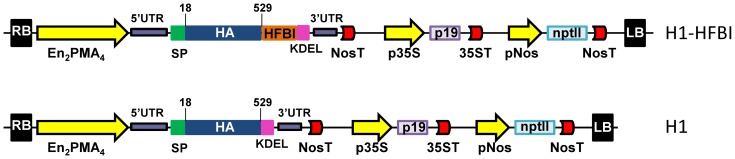
Schematic representation of the pEAQ plasmids used to express H1-HFBI and H1. RB and LB: right and left T-DNA borders, En_2_PMA4: *N. plumbaginifolia* (NpPMA4) promoter reinforced by two copies of the CaMV 35S enhancer (De Muynck et al., 2009); 5′ and 3′UTR: Cowpea Mosaic Virus Untranslated Regions (translational enhancers); SP: *A. thaliana* basic endochitinase signal peptide, HA: hemagglutinin ectodomain (residues 18 to 529), HFBI: hydrophobin I; KDEL: ER retention signal; pNos, NosT: nopaline synthase gene promoter or terminator; p35S, 35ST: Cauliflower Mosaic Virus 35S promoter or terminator; p19: suppressor of RNA silencing; nptII: *neomycin phosphotransferase II* gene.

The sequence encoding the HA ectodomain (codons 18–529) of the A/Texas/05/2009 (H1N1) influenza virus was fused to the *Arabidopsis thaliana* endochitinase signal peptide sequence at the 5′ end, and to the endoplasmic reticulum (ER) retention KDEL sequence at the 3′ end giving H1 (Fig. 1). In addition, the sequence encoding HFBI was fused downstream of the HA ectodomain, via a GGGSGGGS linker, to generate H1-HFBI (Fig. 1) and promote PB formation in the ER. The two resulting sequences were plant codon-optimized (S1 Fig.), and cloned into the binary vector pEAQ-specialK-HT [21], except that the CaMV 35S promoter had been replaced by the En_2_PMA4 promoter, a hybrid promoter made of the *Nicotiana plumbaginifolia PMA4* promoter [22] reinforced by two copies of the CaMV 35S enhancer [23]. We indeed found that the latter allowed higher GFP expression than the former when transiently expressed in *N. benthamiana* leaves (S2 Fig.). The resulting binary vector was electroporated into *Agrobacterium tumefaciens* LBA4404virG, a strain that constitutively expresses virG and allows T-DNA transfer in the absence of the phenolic inducer, acetosyringone [24]. In a preliminary test, the effect of acetosyringone in the infiltration medium on the transient expression of H1-HFBI in *N. benthamiana* leaves was quantified by Western blotting analysis of 16 independent samples collected 6 dpi (S3 Fig.). A 30% decrease of H1-HFBI from 9.7% of total soluble proteins (TSP) to 6.9% TSP was observed in the presence of acetosyringone. Taking into account the HFBI contribution to the H1-HFBI size (11.7%), the actual expression level of H1 was 8.6% TSP and 6.1%, in the presence or absence of AS, respectively.

**Figure 2:**
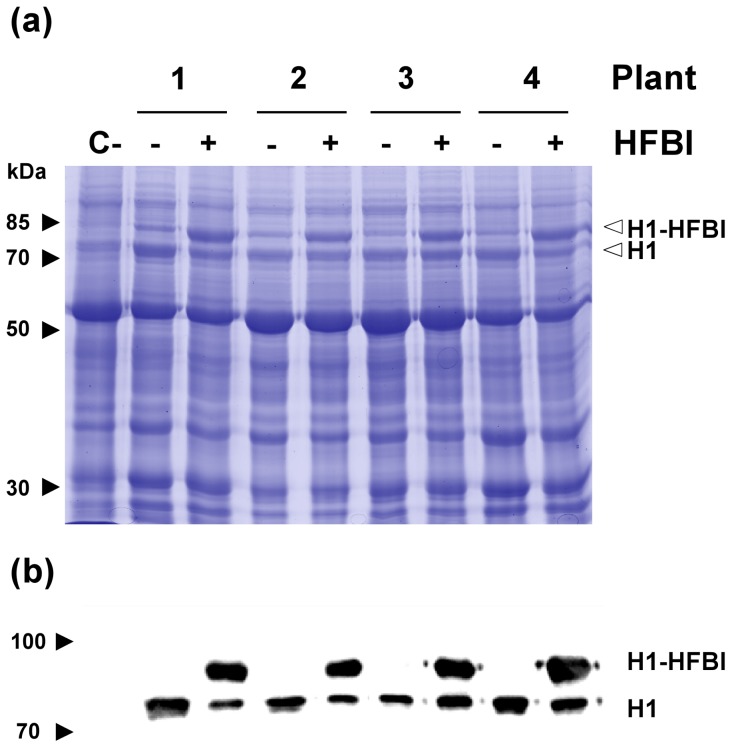
Accumulation of HA fused or not to HFBI. The H1 and H1-HFBI constructs were transiently expressed in *N. benthamiana* leaves. One construct was abaxially infiltrated on one half of the leaf and the other one on the other half. (a) Coomassie-blue stained gel of 30 µg TSP from four representative extracts. TSP from a plant infiltrated with an *A. tumefaciens* strain containing an empty vector was used as a negative control (C-). The bands corresponding to H1-HFBI and H1 are indicated by an arrow. (b) Western blot analysis of 5 µg TSP coming from the same extracts as in (a). The signal was detected with polyclonal anti-influenza A.

*N. benthamiana* leaves were therefore agro-infiltrated with the H1 and H1-HFBI constructs in the absence of acetosyringone, and the expression level of both proteins was analyzed by SDS-PAGE of the soluble protein extracts (Fig. 2a). High expression levels were observed by Coomassie blue staining for both proteins in four individual infiltration experiments. To confirm this observation, Western blotting analysis was performed with polyclonal anti-influenza A antibodies (Fig. 2b). For H1-HFBI, two bands were detected, the major band at an apparent size of 80 kDa and a less abundant band at a size corresponding to untagged H1. This suggests that the H1-HFBI protein was partially cleaved, possibly close to the linker. Regarding their relative abundance, dilution series of three samples containing H1-HFBI were quantified and the resulting signals were compared with the signal of the undiluted samples containing untagged H1 derived from the same leaf (S4 Fig.). This quantification showed that the HFBI fusion enhances the HA expression level by a factor of ∼2.5.

### H1-HFBI accumulates in ER-derived protein bodies

**Figure 3:**
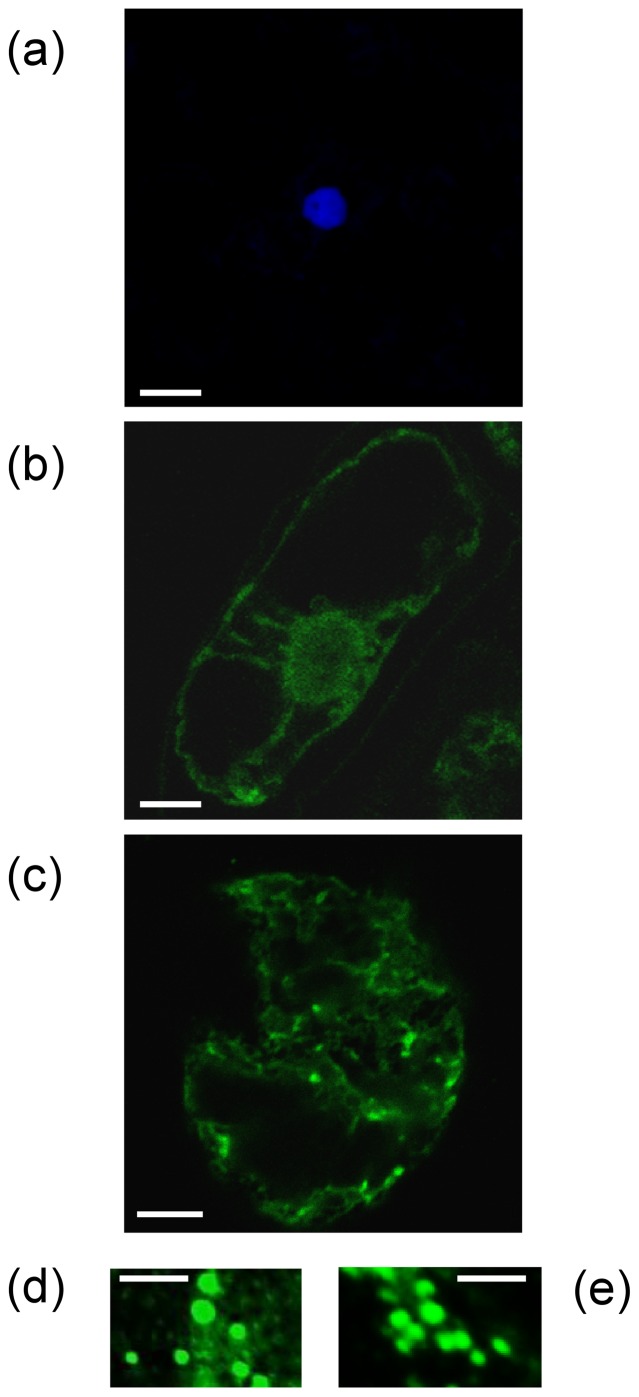
H1-HFBI is found as protein bodies in transgenic *N. tabacum* BY-2 suspension cells. Wild-type cells (a), cells expressing H1 (b) and cells expressing H1-HFBI (c–e) at the exponential phase (3 days after dilution) were submitted to *in situ* immunolocalization as described in the Experimental procedures using an FITC-conjugated anti-influenza H1N1. Bars  = 25 µm (a, b, c) and 5 µm (d, e).

The HFBI fusion is reported to induce the formation of PBs in plants and in plant suspension cells [15]. To determine whether this was the case for H1-HFBI, we sought to visualize such structures in agro-infiltrated leaves by *in situ* immunolocalization, however the negative control already gave too strong of a fluorescence background. We thus transformed *N. tabacum* BY-2 cells with both constructs and performed *in situ* immunolocalization of H1-HFBI and untagged H1 in transgenic cells. Confocal microscopy analysis indicated a signal with a reticulate pattern possibly corresponding to the ER (Fig. 3b). Small spherical particles were detected with a size ranging from 0.2 to 0.5 µm (Figs. 3c and d), reminiscent of protein body structures recently described for GFP-HFBI expressed in *N. tabacum* BY-2 cells [18].

**Figure 4:**
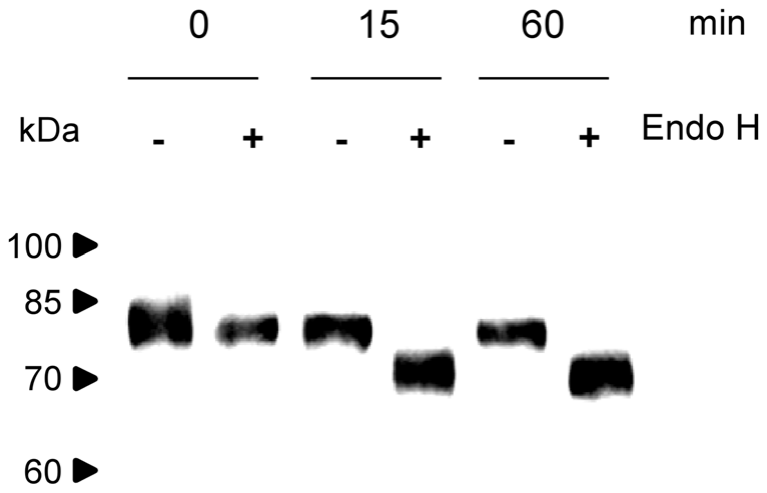
Endoglycosidase H treatment of H1-HFBI. Time course incubation of a leaf TSP extract (5 µg) in the presence (+) or absence (−) of endoglycosidase H (0.2 U/ml). Samples were analyzed by Western blotting after 0, 15, or 60 min of incubation. The signal was detected with polyclonal anti-influenza A antibodies.

To support the ER localization of H1-HFBI, we relied on Endoglycosidase H (EndoH) which specifically cleaves high-mannose N-glycans added in the ER, but not complex N-glycans typically found in glycoproteins that reach the Golgi apparatus. EndoH digestion of a leaf extract with H1-HFBI significantly decreased its apparent size (Fig. 4), indicating that H1-HFBI was glycosylated and efficiently retained in the ER.

### Purification of H1-HFBI protein by aqueous two-phase separation

**Figure 5:**
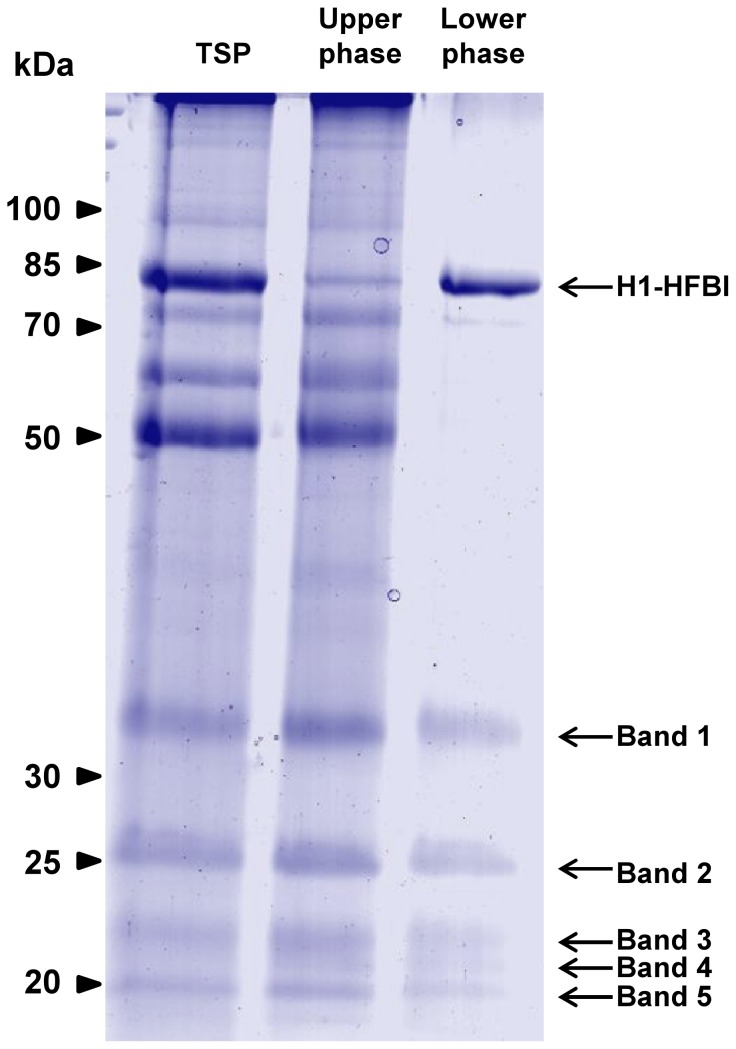
Purification of H1-HFBI by ATPS. A TSP extract from H1-HFBI-expressing leaves was subjected to purification by ATPS as described in the Experimental procedures. Samples (40 µl) of TSP, the upper phase discarded after the first phase separation, and the lower phase recovered after the second phase separation, were analyzed by SDS-PAGE. The identification of bands 1–5 by mass spectrometry is reported in S1 Table.

H1-HFBI was purified from agro-infiltrated leaf extracts by ATPS using Triton X-114 as a surfactant [18], [25]. In ATPS, hydrophobin fusion partners are concentrated within micellar structures and partitioned in a surfactant-rich phase, while the majority of endogenous proteins remain in the aqueous phase and can be discarded. Hydrophobin-fused proteins can be back-extracted by the addition of a non denaturing organic solvent such as isobutanol. To assess the purity of H1-HFBI following ATPS purification, the leaf extract and the ATPS phases were analyzed by SDS-PAGE (Fig. 5). The majority of soluble proteins, including the Rubisco large subunit (∼55 kDa), the most abundant protein in plant leaves, were discarded during the first separation phase. H1-HFBI was found to concentrate in the lower phase with an estimated recovery of about 70% and an overall purity of 50% (as calculated from the signal quantification). The bands corresponding to the five residual contaminating proteins (∼35, 26, 23, 22, and 20 kDa) were excised, trypsin digested, and analyzed by MALDI-TOFTOF mass spectrometry. Their identity, which was confirmed by directly analyzing an ATPS-purified sample by LC-MALDI-TOFTOF mass spectrometry analysis, is given in S1 Table.

### H1-HFBI oligomerizes and exhibits hemagglutination activity

**Figure 6:**
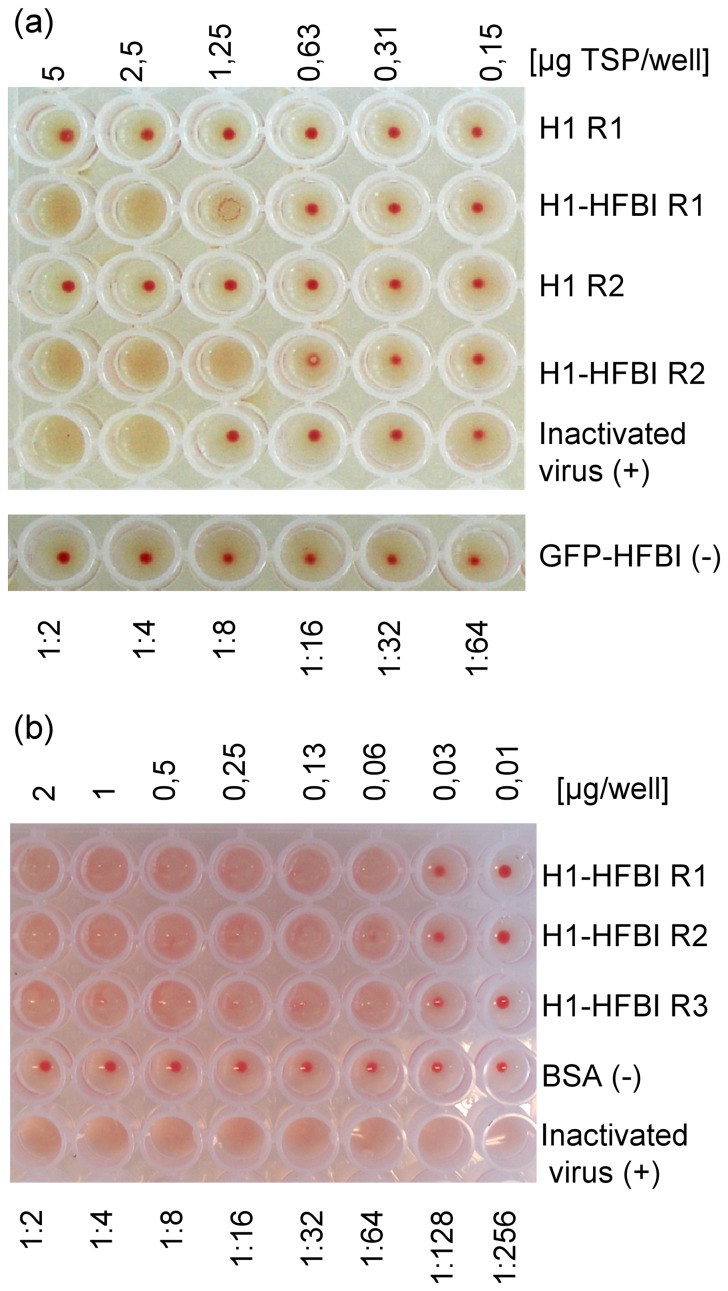
Hemagglutination assay. (a) Hemagglutination assay was performed as indicated in Material and methods using serial two-fold diluted samples of duplicate (R1, R2) TSP fractions extracted from leaves expressing H1-HFBI or untagged H1. The two bottom rows contain inactivated A/Texas/05/2009(H1N1) virus as a positive control or a GFP-HFBI extract as a negative control. (b) Hemagglutination assay using serial two-fold diluted samples of ATPS-purified H1-HFBI (triplicates, R1–R3). The two bottom rows contain bovine serum albumin as a negative control or inactivated A/Texas/05/2009(H1N1) virus as a positive control. The hemagglutination titer (HT) or the amount of hemagglutination units (HAU) was calculated according to the well with the highest dilution giving a complete hemagglutination. This test was also used to quantify inactivated virus concentration in terms of HAU for inhibition assay.

A routine method to demonstrate the biological activity of HA is the hemagglutination assay, which tests its ability to agglutinate red blood cells (RBCs) *in vitro* by binding to sialic acids on surface proteins. We compared cell extracts derived from leaves agro-infiltrated with the H1 or H1-HFBI constructs in a hemagglutination assay and found that the cell extract that contained H1-HFBI was able to agglutinate chicken RBCs, while a cell extract that contained untagged H1 was not (Fig. 6a). A prerequisite for hemagglutination is the formation of oligomeric HA structures that can crosslink cells, and this result indicates that untagged H1 is probably present in a monomeric state while fusion with HFBI allows its oligomerization.

**Figure 7:**
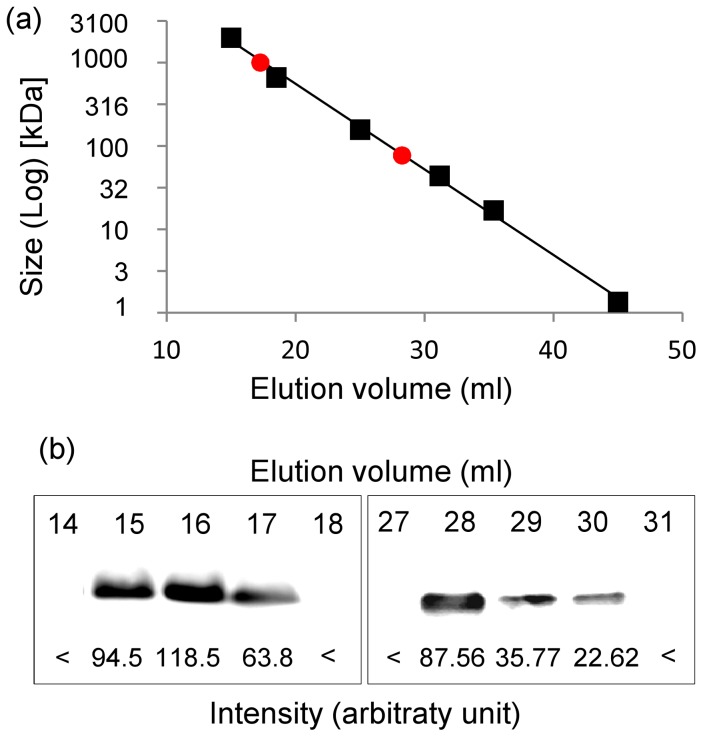
Analysis of the H1-HFBI quaternary structure by size exclusion chromatography. (a) ATPS-purified H1-HFBI (200 µg) was injected onto a Superdex G200 size exclusion column as described in the Experimental procedures. Elution was fractionated in 1 ml aliquots. The logarithmic size of the standards (black square) is plotted according to their elution volume and peaks corresponding to H1-HFBI are indicated (red dot). (b) Fractions eluted at 14 ml to 18 ml and 27 ml to 31 ml were analyzed by Western blotting with an anti-influenza H1N1 and an anti-goat HRP-conjugated secondary antibody, and the signal was quantified using the Kodak image station 4000R (Arbitrary unit; <: below detection level).

As recombinant HA has been observed as monomers, dimers, trimers, and/or high molecular weight oligomers (HMWO) [26], ATPS-purified H1-HFBI was analyzed by size exclusion chromatography to determine its quaternary structure (Fig. 7). The first peak eluted slightly after the void volume of Blue dextran and corresponds to a size higher than the 669 kDa standard. Western blot analysis indicated the presence of H1-HFBI in this peak at an elution volume of 15–17 ml. Elution of the second peak took place between the 158 and 44 kDa standards. Western blot analysis indicated the presence of H1-HFBI at an elution volume of 28–30 ml, which probably corresponds to a monomeric form (the expected size was 68,1 kDa excluding the contribution of glycosylation). We can therefore conclude that H1-HFBI forms both HMWO and monomers. The anti-HA signal detected in the fractions collected for the two forms was quantified after Western blotting and a ratio of HMWO/monomer of approximately 2 was determined.

The ATPS-purified H1-HFBI sample was also subjected to a hemagglutination test with bovine serum albumin (BSA) as a negative control and inactivated A/Texas/05/2009(H1N1) virus as a positive control (Fig. 6b). Hemagglutination was observed with H1-HFBI concentrations of 0.06 µg/well or higher, as well as with the inactivated virus. No hemagglutination was observed with lower H1-HFBI concentrations or with BSA. The HA titer was calculated to be 64 (2^6^). This test demonstrates that hydrophobin-fused HA has retained its receptor binding activity after ATPS purification.

### Immunogenicity of H1-HFBI

**Figure 8:**
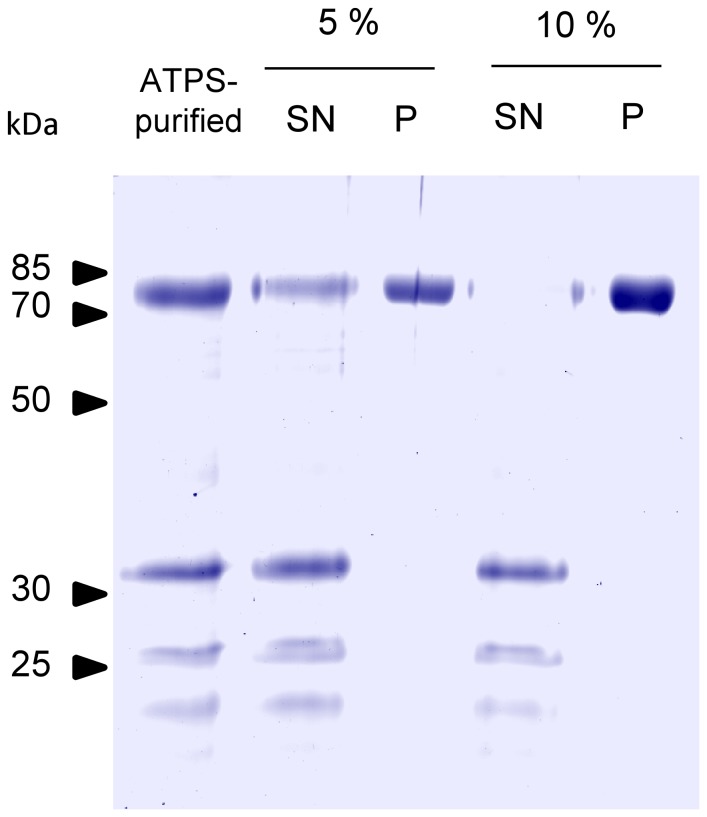
Selective ammonium sulfate precipitation of ATPS-purified H1-HFBI. Solid ammonium sulfate corresponding to 5% or 10% saturation was added to ATPS-purified H1-HFBI samples (4 mg/ml), left for 1 h at 4°C, and centrifuged. The pellet (P) was dissolved in the same volume of PBS as the supernatant volume (SN).

Prior to mice immunization, ATPS-purified H1-HFBI required further purification to remove the remaining contaminants. Varied concentrations of ammonium sulfate were tested for selective precipitation by salting out. Most of H1-HFBI precipitated at 5% saturation of ammonium sulfate while the five contaminating proteins remained in the supernatant (Fig. 8). Increasing the ammonium sulfate concentration to 10% saturation allowed for the complete precipitation of H1-HFBI, which appeared as a single band by SDS-PAGE (Fig. 8), indicating that the protein was purified to apparent homogeneity in the final pellet (for this last step, a recovery of ∼90% with a purity >95% was determined by quantification of the signals). The H1-HFBI precipitate was dissolved in PBS and dialyzed in order to remove excess salts. H1-HFBI exhibited the same hemagglutination activity as shown previously (S5 Fig.) and the same profile as that obtained after size exclusion chromatography (Fig. 7).

**Figure 9:**
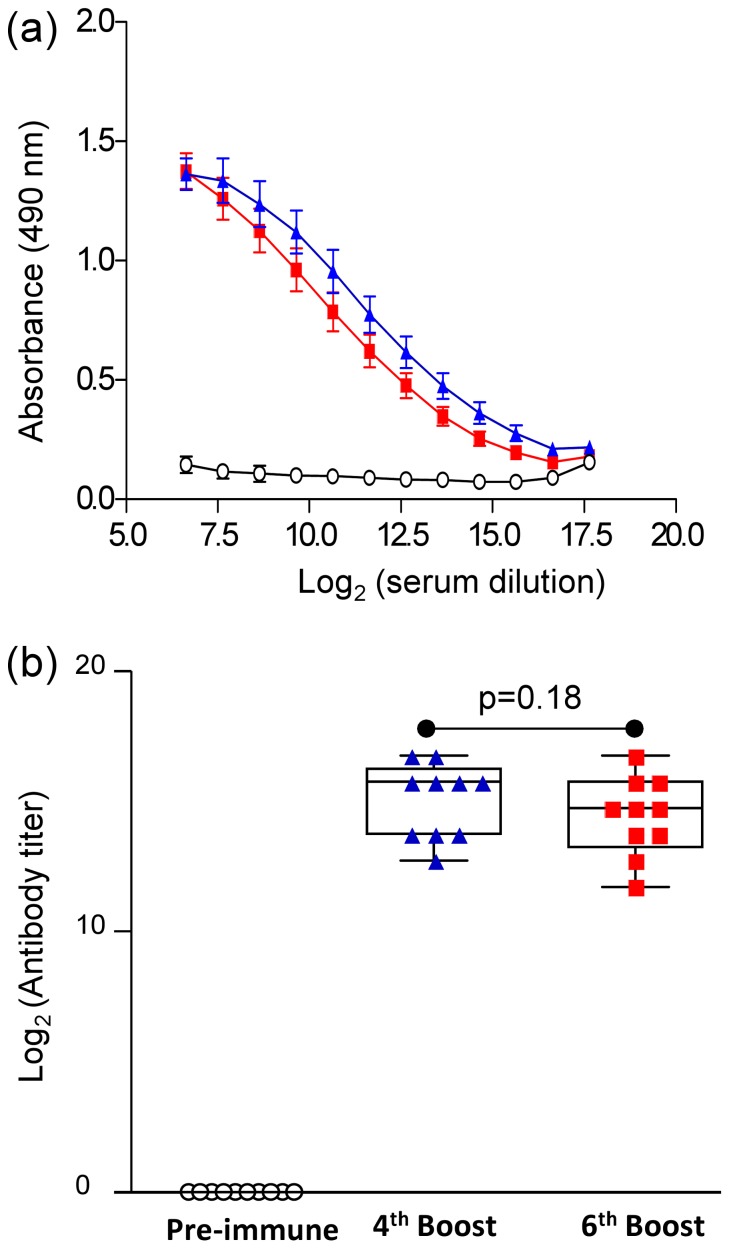
ELISA-based assessment of the immune response of H1-HFBI-immunized mice. (a) Ten mice were immunized with H1-HFBI as indicated in the Experimental procedures. Anti-HA antibodies were assayed by ELISA in the pre-immune sera (open circle) and the sera collected after the 4^th^ (blue triangle) and 6^th^ (red square) boost. Plates were coated with 5 µg/ml of recombinant Influenza A/Texas/05/2009(H1N1) ectodomain expressed in mammalian cells (Sino Biologicals, 11085-V08H). HRP-conjugated anti-mouse secondary antibody was used for detection. HA titer was calculated as the highest dilution giving a signal higher than three times the signal coming from the negative control. (b) Box and whisker analysis of antibody titers obtained after endpoint ELISA titer analysis of the test groups. Each dot represents the antibody titer from an individual mouse. (p-values  = 0.18 (boost 4/boost 6), 1.8.10^−4^ (boost 4/pre-immune), 3.8.10^−4^ (boost 6/pre-immune))

H1-HFBI immunogenicity was evaluated by subcutaneous vaccination of 10 female CD1 mice with 50 µg of purified H1-HFBI formulated with Freund's adjuvant. Pre-immune sera were collected before the first injection, and blood samples were collected after the 4^th^ and the 6^th^ boost. The three samples were analyzed for their ability to recognize a recombinant Influenza A/Texas/05/2009 (H1N1) ectodomain produced in mammalian cells by endpoint titer ELISA (Fig. 9a). Sera of mice immunized with H1-HFBI displayed significantly higher anti-HA titer than the pre-immune sera (p = 3.8.10^−4^). An average HA-specific antibody titer of 25,600 was obtained for the samples collected after the 4^th^ boost, and no statistical difference was observed between the samples collected after the 4^th^ or the 6^th^ boost (p = 0.18) (Fig. 9b).

### The immune response to H1-HFBI results in neutralizing activity

**Figure 10:**
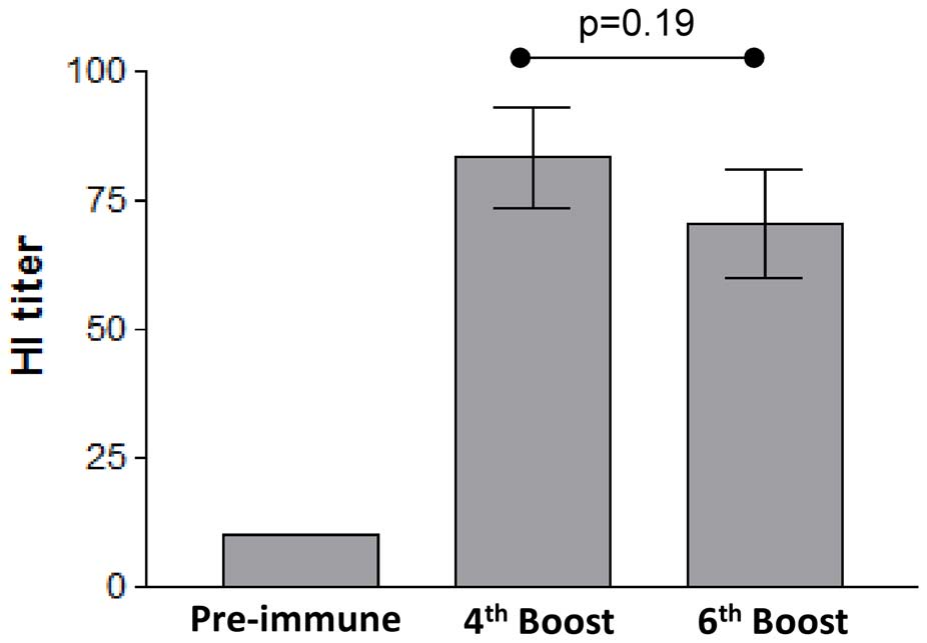
Neutralizing properties of antibodies induced by H1-HFBI. Sera from the ten vaccinated mice were serially 2-fold diluted and incubated with inactivated virus for 30 min, and then RBCs were added. Pre-immune sera were used as a negative control. Hemagglutination inhibition titers were determined. The mean for each test group was calculated, and bars represent SD.

The last step consisted of demonstrating the neutralizing properties of sera from H1-HFBI-vaccinated mice. A reliable test is the hemagglutination inhibition test. Serum samples were incubated with inactivated A/Texas/05/2009 (H1N1) virus, and potential neutralizing antibodies were expected to bind viral receptor binding domains and prevent attachment of the virus to chicken RBCs (Fig. 10). Therefore hemagglutination is prevented when antibodies are present. The highest serum dilution that prevents hemagglutination is designated as the hemagglutination inhibition titer of the serum. None of the pre-immune sera inhibited hemagglutination. Sera collected after the 4^th^ and 6^th^ boost presented a hemagglutination inhibition mean of 83 and 70, respectively. This difference was not statistically significant. We can therefore conclude that H1-HFBI expressed in *N. benthamiana* is able to induce an immune response with neutralizing activity.

## Discussion

Plants have been reported to be an alternative and reliable expression system for seasonal and pandemic influenza vaccines [27]. Transient expression is the most suitable production method regarding influenza pandemics or zoonotic outbreaks. It has been demonstrated that a time period of 3–4 weeks is sufficient to produce a large dose of vaccines [10], [28].

We successfully transiently expressed the hemagglutinin ectodomain of Influenza A/Texas/05/2009 (H1N1) in *N. benthamiana* leaves and obtained a high accumulation level of 8.6% TSP. This was obtained by combining transient transformation, which allows yields well over those obtained in stable transgenic plants, and the pEAQ-HT binary vector, which contains the CPMV UTR's (acting as translation enhancers) as well as the *P19* gene, which prevents silencing [21]. However, three further improvements were made in this work which resulted in enhanced H1 expression. First, the utilization of an *A. tumefaciens* strain which constitutively expressed *virG* made phenolics unnecessary to activate transformation ([24]. As a consequence, the accumulation of H1-HFBI was enhanced by 30% when acetosyringone was omitted from the inoculation medium (S3 Fig.). Second, using the En_2_PMA4 promoter instead of the CaMV 35S promoter was probably an additional asset, since this exchange allowed a 50% increase of GFP expression in transient expression (S2 Fig.). Third, fusion of H1 to HFBI increased expression by ∼2.5 fold (S4 Fig.). The effect of HFBI fusion on HA accumulation is consistent with the 2 fold and 2–3 fold increase of GFP-HFBI reported by Joensuu and co-workers [17] and Gutierrez and co-workers [29], respectively. However, our results contrast with those of Phan and co-workers [19], who showed no yield improvement by fusing HFBI to an H5 ectodomain [19]. This discrepancy might be explained by the weak sequence identity between the H1 and H5 HAs (63%), and the possibility that the effect of HFBI on the accumulation level is protein dependent, as this was observed for ELP fusions [30]. Taken together, the removal of AS, the use of the En_2_PMA4 promoter and the HFBI fusion led to a 330% increase of H1 expression.

Direct comparisons between our data and those previously obtained for the expression of HA in plants is not straightforward for several reasons: the HA origin (virus strain), the portion of HA that is expressed, and the yield calculation (% of TSP or % leaf fresh weight) differ. However, in our hands, since TSP represents ∼6 mg protein/g leaf fresh weight, a H1-HFBI yield of 9.7% TSP is equivalent to ∼600 mg H1-HFBI/kg leaf fresh weight or ∼510 mg H1/kg leaf fresh weight after subtracting the HFBI counterpart. This value exceeds those reported for the transient expression of the complete HA (50 mg/kg; [7]) and is within the same range of HA soluble portions expressed with a launch vector system which involves components of a plant RNA virus (400–1300 mg/kg; [9]). It is also much higher than the 0.05% TSP observed for an H5-HFBI fusion or the 0.5% TSP seen for H5 fused to an elastin-like protein [19]. However, these results were obtained with stable transformation which is known to exhibit reduced performance when compared with transient expression.

Unlike H1, H1-HFBI was located in protein bodies (Fig. 3). This might explain why the HFBI fusion resulted in increased accumulation, as it has been suggested that protein bodies prevent proteolytic degradation [31]. Untagged H5 was partly found in protein bodies, but to a lesser extent than H5-HFBI [19]. HA-HFBI deglycosylation by EndoH strongly supports the ER localization of protein bodies, as EndoH specifically cleaves high-mannose N-glycans added in the ER, but not complex N-glycans typically found in glycoproteins that reach the Golgi. As a decrease of about 10 kDa of H1-HFBI was observed upon EndoH treatment (Fig. 4), and since N-glycosylation increases by ∼2.5 kDa the size of a protein [32], we can guess that four out of the six predicted glycosylation sites [33] are glycosylated.

ATPS purification was shown to be effective for H1-HFBI recovery from plant leaf extracts (Fig. 5). However, a few proteins contaminated the purified fraction. They correspond to abundant TSP proteins and were identified by mass spectrometry (S1 Table). Three out of five identified contaminants are chloroplastic proteins which belong to the oxygen-evolving complex [34]. This complex is one of the three sub-complexes that compose the plant photosystem II, the function of which is to harvest light energy. H1-HFBI without visible contaminants was obtained by combining ATPS purification with differential ammonium sulfate precipitation (Fig. 8). Combining these two approaches also allowed for an increase in the amount of surfactant used during ATPS. As the recovery efficiency is proportional to the concentration of surfactant used [17], using 8 to 10% instead of 4% of Triton X-114 might improve H1-HFBI recovery that was about 70% in this study. The higher surfactant concentration implies a larger volume of the lower phase, but this is unimportant as the following step (ammonium sulfate precipitation) concentrates the purified proteins. This combined purification has the advantage to be scalable, as ATPS has been shown to be efficient up to 20 L [18].

Recombinant hemagglutinin monomers can aggregate in HMWO but this varies according to the viral strain, the expression system, the genetic modifications, and the purification method [26]. Protein aggregates are of interest in vaccination because they are more immunogenic than monomers, although recent concerns about safety were raised [35]. Purified plant-produced H1, H5, and H5-ELP ectodomains have been expressed as monomers [20], [36], but another example from the literature showed plant-produced HMWO [26]. Also, HA from the same influenza strain used in the present study was expressed in *Escherichia coli* as a HMWO [37]. In this study, we observed that the H1 ectodomain alone was probably expressed as monomers, as no hemagglutination was observed while the H1 ectodomain fused to HFBI was expressed as both monomers and oligomers, with a preponderance of the latter. This suggests that the formation of those oligomeric structures is due to HFBI interactions, which is another advantage of this carrier. Yet, this effect may be protein and expression dependent, as it has been shown that HFBI self-interacts at a given concentration [38]. Additionally, in order to increase the oligomeric form and enhance the immunogenicity of H1-HFBI, a trimerization motif such as GCN4-PII could be inserted between HA and HFBI. A fraction of H1-HFBI had a size close to that of H1 (Fig. 2), suggesting that cleavage occurred in the linker region. Changing the latter could further improve the yield of H1-HFBI.

The hemagglutination assay was chosen to assess the biological activity and consequently the proper folding of H1-HFBI. This test was made possible because of the presence of oligomeric structures, whereas monomers are not able to agglutinate RBCs. A positive response using a dose as low as 0.3 µg/ml was obtained. This demonstrates that hydrophobin fusion and the purification method used did not impact the biological activity.

H1-HFBI was shown to induce a significant serum antibody titer in vaccinated mice (Fig. 10). This was demonstrated by ELISA with an HA ectodomain produced in mammalian cells in order to eliminate a potential response coming from anti-hydrophobin antibodies and to confirm the specificity of the induced anti-HA antibodies. H1-HFBI is also expected to be protective in mice, as the calculated hemagglutination inhibition titer was 83, while the minimal titer required for a vaccine to be protective in humans is 40 [39]. Blood samples were collected after the 4^th^ and the 6^th^ booster immunization but no significant differences were observed between the two samples. Regarding pandemics, when part of the population is immunologically naïve to the emerging viral strain, two vaccine doses may be required, while one dose should be enough for people with preexisting immunity against the virus lineage. Therefore, four booster immunizations is too high and a dose-ranging study has to be performed to investigate the potential of this vaccine candidate. This study should also include different adjuvants such as Qiul A or AbISCO. One might wonder whether injection of the fungal hydrophobin to animals and humans could trigger deleterious effects. Hydrophobins have a nontoxical nature [40] and prevent immune recognition of fungal spores, suggesting that they are not immunogenic [41]. This suggests that hydrophobin fusion is safe for antigen design even though further investigation must be performed. As an exploratory experiment, the presence of anti-HFBI antibodies in a vaccinated mouse serum was assessed by Western blotting of HFBI fused to another protein, the Green Fluorescent Protein (GFP) as well as of unfused GFP as a control, both expressed in *N. benthamiana* (S6 Fig.). The serum did not show HFBI recognition. However, Nakari-Setälä and colleagues were able to produced anti-HFBI antibodies by immunization of rabbits [42], indicating that the protein might be immunogenic. Therefore, further investigations are required to determine the HFBI immunogenicity and its impact on the immunogenicity of the fused protein. In case of a negative impact or the formation of anti-HFBI antibodies that might be deleterious for the patient, the addition of a proteolytic cleavage site between H1 and HFBI could be considered. This cleavage could also be useful to remove a potential trimerization motif added to stabilize the oligomeric form into a more homogenous product.

Many efforts have been carried out to find an alternative to the current egg-based vaccine technology. In this study, we investigated a hydrophobin fusion to a recombinant hemagglutinin ectodomain. This fusion was shown to enhance the accumulation level and to allow rapid, easy, and scalable purification while the fused protein remained biologically active, was immunogenic, and induced neutralizing antibodies in mice. Transient expression of H1-HFBI is therefore a promising approach to produce seasonal and/or pandemic influenza vaccines.

## Experimental Procedures

### Ethics statement

The experiments, maintenance and care of mice complied with the guidelines of the European Convention for the Protection of Vertebrate Animals used for Experimental and other Scientific Purposes (CETS n° 123). The protocol was approved by the Committee on the Ethics of Animal Experiments of the University of Liège, Belgium (Permit Number: 06-594). All efforts were made to minimize suffering.

### Materials

*Escherichia coli* Top 10 or *Agrobacterium tumefaciens* LBA4404virG (van der Fits et al., 2000) were used for cloning experiments and plant transformation, respectively. *Nicotiana benthamiana* plants were grown in compost in a growth chamber with 16 h of light (200 µmol.s^−1^.m^−2^) at 25°C and 8 h of darkness at 19°C.

*Nicotiana tabacum* cv. Bright yellow-2 (BY-2) [43] suspension cells were grown in MS medium (30 g/l sucrose, 4.4 g/l Murashige and Skoog medium, 0.2 g/l KH_2_PO_4_, 50 mg/l myo-inositol, 2.5 mg/l thiamine, 0.2 mg/l 2-4D, pH 5.8 (KOH)) at 25°C under dark light conditions with agitation at 90 rpm. The cultures were diluted 1∶20 in fresh medium every week. Transformed cells were grown in medium supplemented with 100 µg/ml of kanamycin.

### Construction of the H1 and H1-HFBI expression vectors

The pEAQspecialK-HT plasmid [21] was used as an expression vector. The initial p35S promoter was replaced by the *Nicotiana plumbaginifolia* H^+^-ATPase PMA4 promoter reinforced with two copies of the 35S enhancer [23]. The DNA sequence corresponding to codons 1 to 566 of the influenza A/Texas/05/2009(H1N1) *HA* gene was optimized for plant expression and synthesized by GenScript (Piscataway, USA). The sequence encoding the HA ectodomain (codons 18–529) was amplified from this sequence using the primers HA-Chit (5′ TATCCTCGGCCGAAGATACCCTCTGCATTGG 3′) and HA-HFBIR (5′ CGAGTGAACCACCACCCTGATAGATCCTGGTACTC 3′). This was fused by overlap extension PCR to the signal peptide sequence (codons 1 to 21) of the *Arabidopsis thaliana* basic endochitinase (Accession number: P19171) amplified from pSK-chit-OspA [44] using the primers chitAgeI (5′AACACCGGTATGAAGACTAATCTTTTTCTC 3′, AgeI site underlined) and Chit-HA (5′ CCAATGCAGAGGGTATCTTCGGCCGAGGATAATGAT 3′). The resulting chit-HA fragment was cloned into the pGEM-T Easy vector and sequenced. A DNA sequence corresponding to a GGGSGGGS linker, codons 23 to 97 of the *HFBI* gene from *Trichoderma reseei* (P52754), a GGGG linker, and the KDEL ER-retrieval motif was optimized for plant expression and synthesized by GenScript (Piscataway, USA). The sequence was amplified by PCR with the primers HA-HFBIF (5′ GAGTACCAGGATCTATCAGGGTGGTGGTTCACTCG 3′) and HFBIXhoI (5′ TTGCTCGAGTCATAACTCATC3′, XhoI underlined). The HFBI-KDEL amplicon was fused by overlap extension PCR to the chit-HA fragment. The resulting PCR product was introduced into pEAQ-HT using AgeI/XhoI to generate the pEAQ-H1-HFBI binary plasmid.

The untagged *H1* gene construct was obtained by PCR from the H1-HFBI construct using the primers chitAgeI and KDELXhoI (5′ TTGCTCGAGCTGATAGATCCTGGTACTCTC 3′, XhoI underlined) and cloned into pEAQ-HT to give pEAQ-H1-HT. The nucleotide and amino acid sequences of H1-HFBI and H1 are displayed in S1 Fig.

### Plant transient transformation

The pEAQ-H1-HT and pEAQ-H1-HFBI-HT binary plasmids were introduced into *A. tumefaciens* LBA4404 virG by electroporation. The *A. tumefaciens* strains were grown overnight at 28°C in 2YT medium (1.6% bacto-tryptone, 1% bacto-yeast-extract, 0.1% glucose, 0.02% MgSO_4_) supplemented with 20 µg/ml rifampicin, 40 µg/ml gentamycin, and 50 µg/ml kanamycin. The cells were harvested by centrifugation (5,000 *g*, 5 min, 15°C), washed three times in infiltration medium (10 mM MES monohydrate, 10 mM MgCl_2_, pH 5.3 (KOH)), and resuspended in infiltration medium at a final OD_600_ of 0.6. *N. benthamiana* leaves were then infiltrated on the abaxial side through the stomata using a syringe. The plants were incubated for 6 days under routine culture conditions.

### *N. tabacum* BY-2 cell stable transformation

*N. tabacum* BY-2 suspension cells were transformed by co-cultivation with *A. tumefaciens* as described previously [44]. Transgenic calli were selected on MS medium supplemented with 100 µg/ml kanamycin.

### Protein electrophoresis and Western blotting

The protein content of the different samples was determined using the Bradford method [45].

Proteins were solubilized in SDS loading buffer (80 mM Tris-HCl, pH 6.8, 2% SDS, 10% glycerol, 0.005% bromophenol blue, 60 mM DTT, 1 mM PMSF, and 1 µg/ml each of leupeptin, aprotinin, antipain, chymostatin, pepstatin), boiled for 5 min, and separated by SDS-PAGE (10% polyacrylamide gel).

For colloidal Coomassie blue staining, gels were incubated at room temperature for 2 h in fixation solution (50% ethanol, 2% phosphoric acid), washed three times with water, stained overnight in staining solution (34% methanol, 17% ammonium sulfate, 3% phosphoric acid, 700 mg/l of Coomassie Brilliant Blue G-250 (Serva, Heidelberg, Germany), and destained in water.

For Western blotting, proteins were electrotransferred onto a polyvinylidene fluoride membrane, then the membrane was saturated, incubated first with goat anti-influenza A (1∶1,000, OBT1551, Abdserotec, UK) for 1 h at room temperature, and incubated a second time with anti-goat HRP-conjugated (1∶10,000, A5420, Sigma, St-Louis, MO) for 1 h at room temperature. The membrane was incubated for 2 min with Lumi-light (Roche, Switzerland) and the signals were quantified using the Kodak Image Station 4000R (Eastman Kodak company, Rochester, NY).

To obtain a rough estimation of the expression level of recombinant H1 and H1-HFBI proteins, an immunoblotting technique was applied using the extracellular domain of a recombinant Influenza A/Texas/05/2009(H1N1) hemagglutinin produced in human cells (11085-V08H, Sino Biologicals, China) as a standard. The soluble fraction obtained after homogenization of agro-infiltrated leaves was serially diluted to obtain band intensities that were similar to the band intensity of the standard protein used at varied amounts (50, 100, 200, 500 ng). Band intensities were quantified using Kodak Image Station 4000R software. For H1-HFBI quantification, 16 independent samples were analyzed.

### Endoglycosidase H treatment

Leaf protein (5 µg) samples containing H1-HFBI were diluted in 50 mM sodium citrate pH 5.5 (HCl), 0.5 mM PMSF. Then, 0.2 U/ml Endoglycosidase H (Roche) was added. After incubation at 37°C for 0, 15, or 60 min, the reaction was stopped by the addition of SDS loading buffer.

### *In situ* immunolocalization

Three-day-old transgenic *N. tabacum* BY-2 cell cultures (1.6 ml) were harvested by centrifugation at 900 *g* for 10 min (Mikro 20, Hettich Zentrifugen), and the location of H1-HFBI and untagged H1 were identified by *in situ* immunolocalization as described in [46] using FITC-conjugated anti-influenza (1∶20 dilution; Ab20388, Abcam, UK).

### H1-HFBI extraction and purification

Agro-infiltrated leaves were frozen in liquid nitrogen and homogenized using a mortar and a pestle. The resulting powder was then resuspended in 10 volumes of ice-cold PBS (137 mM NaCl, 2.7 mM KCl, 10 mM Na_2_HPO_4_, 2 mM KH_2_PO_4_) supplemented with 1 mM PMSF, and homogenized in a potter or a shaker for small or large volumes, respectively. The homogenate was clarified by centrifugation at 15,000 *g* for 20 min at 4°C. Membrane proteins were discarded by ultracentrifugation at 125,000 *g* for 30 min at 4°C.

For ATPS, TSP were pre-warmed in a water bath until the temperature reached 28°C. TSP were then mixed vigorously with 4% (w/v) of Triton X-114 and introduced into a separation funnel. The two phases were allowed to separate for 15 min at 28°C. The bottom phase was recovered and detergent was removed by the addition of isobutanol (10 times the Triton X-114 volume). After centrifugation at 5,000 *g* for 5 min at room temperature, the new bottom phase containing H1-HFBI was recovered.

ATPS-purified H1-HFBI was further purified by adding solid ammonium sulfate up to 5% or 10% of the final concentration. The solution was then stirred for 1 h at 4°C and centrifuged at 20,000 *g* for 15 min at 4°C. The supernatant was discarded and the remaining pellet was dissolved in PBS.

### Protein analysis by mass spectrometry

The bands corresponding to proteins were excised from the gel, treated with trypsin, and analyzed by MS/MS, as described in [47] and as detailed in S1 Table.

### Gel filtration chromatography

For size exclusion chromatography, a Superdex G200 column (GE Healthcare, UK) (10×300 mm) was used coupled to an Äkta Explorer (GE Healthcare) purification system. After column equilibration with PBS, 100 µl of gel filtration standards (Bio-Rad, #151-1901) were injected and a flow rate of 1 ml/min was applied until the last standard was eluted. Then, a 500 µl ATPS-purified sample was applied to the column under the same conditions. Effluents were collected in 1 ml fractions.

### Hemagglutination assay and hemagglutination inhibition assay

Functional activity of H1-HFBI was evaluated using a hemagglutination assay according to standard procedures [48]. Recombinant H1-HFBI was diluted to a final concentration of 4 µg/ml in PBS, and 50 µl aliquots were serially two-fold diluted in U-bottom 96-well plates. After the addition of 50 µl of 1% (w/v) RBCs, the plates were incubated for 30 min at 20°C. Bovine serum albumin was used as a negative control.

The hemagglutination inhibition assay was based on standard procedures as well [46]. Influenza A/Texas/05/2009 virus purified from allantoïc fluid and formol-inactivated was diluted to a final concentration of 8 HAU/50 µl after quantification by a hemagglutination assay. Sera from immunized mice were diluted with three volumes of Cholera filtrate containing Receptor Destroying Enzyme (Sigma, C8772-1VL) and incubated for 16 h at 37°C, then heat inactivated for 30 min at 56°C. Two-fold dilution of inactivated sera was followed by incubation with 4 HAU of A/Texas/05/2009 virus for 30 min at 20°C. Chicken erythrocytes (1%) were added and incubated for an additional 40 min at 20°C. The HAI titer was calculated as the reciprocal of the highest dilution that produced complete hemagglutination inhibition.

### Mice immunization

A group of 10 8 week-old female CD1 mice were blood sampled before vaccination. The mice were then vaccinated intraperitoneally with 50 µg of purified H1-HFBI every two weeks for a total of 6 hyperimmunizations. The vaccine was formulated with Complete Freund's adjuvant for the first two immunizations, and with Incomplete Freund's adjuvant for the booster immunizations. Blood samples were collected after 4 boosts using a slight incision in the mice tails. Two weeks after the 6^th^ boost, the mice were killed humanly by an overdose of pentobarbital and an exsanguination. The sera collected were used for indirect ELISA and hemagglutination inhibition assay.

### Indirect ELISA

Microtiter plates were coated with 100 µl/well of 5 µg/ml (in PBS) of extracellular domain of recombinant Influenza A/Texas/05/2009 (H1N1) hemagglutinin produced in human cells (Sino Biologicals, 11085-V08H) and incubated for 16 h at 4°C. The plates were then washed three times in PBST (0.1% Tween 20 in PBS) and saturated for 1 h at room temperature with 200 µl/well of PBS supplemented with 5% dried non-fat milk. After three additional washing steps, 100 µl of a 1∶100 dilution of sera from the immunized mice were serially two-fold diluted and incubated for 90 min at 37°C. The plates were then washed three times with PBST and incubated for 1 h at room temperature with 100 µl/well of a 1∶10,000 dilution of anti-mouse HRP-conjugated IgG (Biognost Millipore, AP308P). After four washes, 100 µl/well of o-phenylenediamine peroxidase substrate (Sigma) in citrate buffer (0.05 M Na_2_HPO_4_, 0.025 M citric acid) was added. The reaction was stopped after 15 min with 50 µl of 1 M H_2_SO_4_ and the absorbance measured at 490 nm (Model 550, Microplate Reader; Bio-Rad, Hercules, CA).

## Supporting Information

S1 Fig **Nucleotide and amino acid sequence of H1-HFBI.** Purple: *A. thaliana* endochitinase signal peptide, White: Influenza A/Texas/05/2009 (H1N1) ectodomain, Red: linker, Yellow: HFB I, Green: KDEL retention signal.
(DOCX)

S2 Fig **Transient GFP expression comparison in** ***N. benthamiana*** **leaves between two strong transcriptional promoters.** Leaves were infiltrated with an *A. tumefaciens* strain containing the pEAQ-HT vector with the *GFP* gene driven by the p35S or En_2_PMA4 promoter. A leaf transformed with an empty pEAQ-HT vector was used as a negative control. A TSP fraction was prepared at 6 dpi. (a) Twenty µg of TSP were analyzed by SDS-PAGE and the gel was stained with colloidal blue. The large Rubisco subunit is indicated (*), and the GFP is indicated by an arrow. (b) The GFP content of six independent samples (50 µg TSP) for each promoter was quantified by fluorimetry (excitation at 395 nm and emission at 508 nm).
(PDF)

S3 Fig **Impact of acetosyringone in the infiltration medium on HA-HFBI accumulation.** Following agroinfiltration with the H1-HFBI construct in the presence (+) or absence (−) of acetosyringone in the infiltration medium, total soluble proteins (TSP) were extracted at 6 dpi and the band corresponding to H1-HFBI was quantified by Western blotting. (n = 16, bars  =  SD, p-value  = 0.0011).
(PDF)

S4 Fig **Relative quantification of H1-HFBI and H1 accumulation.** The H1 and H1-HFBI constructs were transiently expressed in *N. benthamiana* leaves. One construct was infiltrated on one half of the leaf and the other one on the other half. The H1-HFBI TSP fractions (1 µg) were diluted two, three, or four times and compared to undiluted H1 TSP fractions (1 µg) by Western blot analysis. Values obtained after quantification with the Kodak Image Station 4000R are displayed below each sample in arbitrary units.
(DOCX)

S5 Fig **Hemagglutination assay of purified HA-HFBI.** Hemagglutination assay was performed as indicated in the Experimental procedures using serial two-fold diluted samples of dissolved ammonium sulfate precipitate of H1-HFBI (duplicate R1, R2). The upper row contains BSA as a negative control.
(PDF)

S6 Fig **Evaluation of anti-HFBI antibodies in immunized mouse serum.** Samples containing H1-HFBI, GFP-HFBI or GFP (15 µg TSP) transiently expressed in *N. benthamiana* were analyzed by Western blotting using a 1∶200 diluted serum from mouse 6 immunized with H1-HFBI and a 1∶5000 dilution of a HRP-conjugated anti-mouse secondary antibody. The membrane was then stripped in 0.4N NaOH for 3 min and then incubated with a polyclonal anti-GFP antibody and a polyclonal anti-rabbit secondary antibody. Samples coming from a leaf infiltrated with an empty vector and a commercial recombinant H1 expressed in mammalian cells (rHA(+)) were used as negative and positive controls, respectively. Note thatadditional bands were detected with the mouse serum at a size similar to unfused GFP. They probably do not correspond to GFP as the fused GFP-HFBI is not recognized.
(DOCX)

S1 Table **Identification of the proteins contaminating the ATPS-purified sample^1^.** ^1^Method: The acquired spectra were analyzed using the Applied Biosystems GPS Explorer (version 3.6) and the Matrix Science MASCOT algorithm in the NCBI *N. benthamiana* database and the NCBI *N. benthamiana* EST database, as described in Duby et al. (2010). Reverse phase separation of peptides was completed on an Ultimate 3000 chromatography chain (ThermoFisher Scientific) using a C18 PepMap 100 analytical column (150 mm, 3 µm i.d., 100 Å), (ThermoFisher Scientific). Previously the sample was dissolved in 0.025% (v/v) TFA and 5% (v/v) ACN and desalted using a C18 Pep Map 100 pre-column (10 mm, 5 µm i.d., 100 Å). Peptides were backflushed onto the analytical column with a flow rate of 300 nL/min by a 180 min linear gradient from 8 to 76% (v/v) ACN in water containing 0.1% (v/v) TFA in buffer A and 0.085% (v/v) TFA in buffer B. The eluted peptides were mixed with a-cyano-4-hydrocinnamic acid (4 mg/mL in 70% ACN/0.1% TFA) and spotted directly onto a MALDI target using a Probot system (ThermoFisher Scientific). ^2^The band numbers correspond to those annotated in Fig. 5.
(PDF)
