# Supplementary material for: Hydrophobin Fusion of an Influenza Virus Hemagglutinin Allows High Transient Expression in Nicotiana benthamiana, Easy Purification and Immune Response with Neutralizing Activity
Source: PLoS One. 2014 Dec 26;9(12):e115944. doi: 10.1371/journal.pone.0115944 (PMC4277400; doi:10.1371/journal.pone.0115944)
Supplement: S1 Fig — Nucleotide and amino acid sequence of H1-HFBI. Purple: A. thaliana endochitinase signal peptide, White: Influenza A/Texas/05/2009 (H1N1) ectodomain, Red: linker, Yellow: HFB I, Green: KDEL retention signal. (DOCX) [file pone.0115944.s001.docx]

| 1 |  | M__K__T__N__L__F__L__F__L__I__F__S__L__L__L__S__L__S__S__A__ |
| --- | --- | --- |
| 1 |  | ATGAAGACTAATCTTTTTCTCTTTCTCATCTTTTCACTTCTCCTATCATTATCCTCGGCC |
| 21 |  | E__D__T__L__C__I__G__Y__H__A__N__N__S__T__D__T__V__D__T__V__ |
| 61 |  | GAAGATACCCTCTGCATTGGATACCACGCAAACAACTCTACCGATACTGTTGATACTGTG |
| 41 |  | L__E__K__N__V__T__V__T__H__S__V__N__L__L__E__D__K__H__N__G__ |
| 121 |  | CTTGAAAAGAATGTTACAGTGACCCATTCTGTTAATCTTTTGGAGGATAAGCACAATGGT |
| 61 |  | K__L__C__K__L__R__G__V__A__P__L__H__L__G__K__C__N__I__A__G__ |
| 181 |  | AAACTTTGTAAACTCAGAGGAGTGGCTCCTTTACATCTCGGAAAGTGTAATATTGCAGGT |
| 81 |  | W__I__L__G__N__P__E__C__E__S__L__S__T__A__S__S__W__S__Y__I__ |
| 241 |  | TGGATCTTAGGAAACCCAGAATGCGAGTCTCTCTCAACAGCTTCTTCATGGTCATACATC |
| 101 |  | V__E__T__S__S__S__D__N__G__T__C__Y__P__G__D__F__I__D__Y__E__ |
| 301 |  | GTTGAAACTAGTTCTTCAGATAATGGTACATGTTATCCTGGAGATTTCATTGATTACGAA |
| 121 |  | E__L__R__E__Q__L__S__S__V__S__S__F__E__R__F__E__I__F__P__K__ |
| 361 |  | GAGTTAAGAGAGCAACTCAGTTCTGTTTCAAGTTTTGAAAGGTTCGAGATTTTTCCTAAG |
| 141 |  | T__S__S__W__P__N__H__D__S__N__K__G__V__T__A__A__C__P__H__A__ |
| 421 |  | ACATCTTCATGGCCAAATCATGATTCTAACAAAGGAGTTACCGCTGCATGCCCTCACGCT |
| 161 |  | G__A__K__S__F__Y__K__N__L__I__W__L__V__K__K__G__N__S__Y__P__ |
| 481 |  | GGTGCAAAGTCATTCTATAAGAATCTTATCTGGTTGGTGAAGAAAGGAAACAGTTACCCA |
| 181 |  | K__L__S__K__S__Y__I__N__D__K__G__K__E__V__L__V__L__W__G__I__ |
| 541 |  | AAGCTTAGTAAATCTTACATTAATGATAAGGGAAAAGAAGTTCTTGTGTTGTGGGGTATC |
| 201 |  | H__H__P__S__T__S__A__D__Q__Q__S__L__Y__Q__N__A__D__A__Y__V__ |
| 601 |  | CATCACCCATCAACTAGTGCTGATCAACAGTCTTTGTATCAAAACGCTGATGCATACGTT |
| 221 |  | F__V__G__S__S__R__Y__S__K__K__F__K__P__E__I__A__I__R__P__K__ |
| 661 |  | TTCGTGGGAAGTTCTAGATACTCTAAGAAGTTTAAGCCTGAAATCGCAATTAGACCAAAA |
| 241 |  | V__R__D__Q__E__G__R__M__N__Y__Y__W__T__L__V__E__P__G__D__K__ |
| 721 |  | GTTAGGGATCAGGAGGGTAGGATGAATTATTACTGGACACTTGTGGAACCTGGAGATAAG |
| 261 |  | I__T__F__E__A__T__G__N__L__V__V__P__R__Y__A__F__A__M__E__R__ |
| 781 |  | ATTACCTTCGAGGCTACTGGTAATTTGGTTGTGCCAAGATACGCTTTTGCTATGGAAAGG |
| 281 |  | N__A__G__S__G__I__I__I__S__D__T__P__V__H__D__C__N__T__T__C__ |
| 841 |  | AACGCAGGTAGTGGAATTATCATTTCTGATACTCCTGTTCATGATTGTAATACTACATGC |
| 301 |  | Q__T__P__K__G__A__I__N__T__S__L__P__F__Q__N__I__H__P__I__T__ |
| 901 |  | CAAACCCCAAAAGGTGCTATTAATACTTCTCTTCCTTTCCAGAACATCCACCCAATCACA |
| 321 |  | I__G__K__C__P__K__Y__V__K__S__T__K__L__R__L__A__T__G__L__R__ |
| 961 |  | ATTGGAAAGTGTCCTAAATATGTTAAGTCAACAAAGCTCAGACTTGCTACTGGTCTTAGG |
| 341 |  | N__V__P__S__I__Q__S__R__G__L__F__G__A__I__A__G__F__I__E__G__ |
| 1021 |  | AATGTGCCATCTATTCAGTCAAGAGGTTTATTCGGAGCTATTGCAGGTTTTATTGAAGGT |
| 361 |  | G__W__T__G__M__V__D__G__W__Y__G__Y__H__H__Q__N__E__Q__G__S__ |
| 1081 |  | GGATGGACTGGTATGGTTGATGGTTGGTATGGATACCATCACCAAAATGAGCAGGGTTCA |
| 381 |  | G__Y__A__A__D__L__K__S__T__Q__N__A__I__D__E__I__T__N__K__V__ |
| 1141 |  | GGATACGCTGCAGATTTGAAGAGTACACAAAACGCTATTGATGAAATCACCAATAAGGTT |
| 401 |  | N__S__V__I__E__K__M__N__T__Q__F__T__A__V__G__K__E__F__N__H__ |
| 1201 |  | AACTCTGTGATTGAGAAGATGAATACTCAGTTCACAGCAGTTGGAAAGGAGTTTAATCAT |
| 421 |  | L__E__K__R__I__E__N__L__N__K__K__V__D__D__G__F__L__D__I__W__ |
| 1261 |  | CTTGAAAAGAGGATCGAGAATTTGAACAAGAAGGTTGATGATGGTTTTCTTGATATCTGG |
| 441 |  | T__Y__N__A__E__L__L__V__L__L__E__N__E__R__T__L__D__Y__H__D__ |
| 1321 |  | ACTTATAATGCTGAATTACTCGTGCTTTTGGAAAACGAGAGAACATTGGATTACCATGAT |
| 461 |  | S__N__V__K__N__L__Y__E__K__V__R__S__Q__L__K__N__N__A__K__E__ |
| 1381 |  | TCTAACGTTAAGAACCTTTACGAGAAAGTGAGGTCACAATTGAAGAATAACGCTAAGGAA |
| 481 |  | I__G__N__G__C__F__E__F__Y__H__K__C__D__N__T__C__M__E__S__V__ |
| 1441 |  | ATCGGTAATGGTTGCTTTGAGTTCTATCACAAGTGTGATAACACTTGCATGGAAAGTGTT |
| 501 |  | K__N__G__T__Y__D__Y__P__K__Y__S__E__E__A__K__L__N__R__E__E__ |
| 1501 |  | AAAAACGGAACATATGATTACCCTAAGTATTCTGAAGAGGCTAAACTTAATAGAGAAGAG |
| 521 |  | I__D__G__V__K__L__E__S__T__R__I__Y__Q__G__G__G__S__G__G__G__ |
| 1561 |  | ATTGATGGTGTTAAGTTAGAGAGTACCAGGATCTATCAGGGTGGTGGTTCAGGTGGTGGT |
| 541 |  | S__S__N__G__N__G__N__V__C__P__P__G__L__F__S__N__P__Q__C__C__ |
| 1621 |  | TCAAGTAACGGTAATGGTAATGTGTGCCCTCCAGGTTTATTTTCTAATCCTCAATGTTGC |
| 561 |  | A__T__Q__V__L__G__L__I__G__L__D__C__K__V__P__S__Q__N__V__Y__ |
| 1681 |  | GCAACCCAGGTTTTAGGTCTCATTGGACTTGATTGTAAGGTTCCATCACAAAACGTGTAC |
| 581 |  | D__G__T__D__F__R__N__V__C__A__K__T__G__A__Q__P__L__C__C__V__ |
| 1741 |  | GATGGTACTGATTTCAGAAACGTGTGCGCAAAAACAGGAGCTCAGCCTTTGTGTTGCGTT |
| 601 |  | A__P__V__A__G__Q__A__L__L__C__Q__T__A__V__G__A__G__G__W__S__ |
| 1801 |  | GCTCCAGTGGCAGGTCAAGCTCTTTTGTGTCAGACAGCTGTTGGAGCAGGAGGTTGGTCA |
| 621 |  | H__P__Q__F__E__K__G__G__K__D__E__L__*__ |
| 1861 |  | CACCCACAGTTTGAGAAAGGAGGAAAAGATGAGTTATGA |

Figure S1. Nucleotide and amino acid sequence of H1-HFBI

Purple: *Arabidopsis thaliana* endochitinase signal peptide, White: Influenza A/Texas/05/2009 (H1N1) ectodomain, Red: linker, Yellow: HFB I, Green: KDEL retention signal.
