# Supplementary material for: Hydrophobin Fusion of an Influenza Virus Hemagglutinin Allows High Transient Expression in Nicotiana benthamiana, Easy Purification and Immune Response with Neutralizing Activity
Source: PLoS One. 2014 Dec 26;9(12):e115944. doi: 10.1371/journal.pone.0115944 (PMC4277400; doi:10.1371/journal.pone.0115944)
Supplement: S2 Fig — Transient GFP expression comparison in N. benthamiana leaves between two strong transcriptional promoters. Leaves were infiltrated with an A. tumefaciens strain containing the pEAQ-HT vector with the GFP gene driven by the p35S or En2PMA4 promoter. A leaf transformed with an empty pEAQ-HT vector was used as a negative control. A TSP fraction was prepared at 6 dpi. (a) Twenty µg of TSP were analyzed by SDS-PAGE and the gel was stained with colloidal blue. The large Rubisco subunit is indicated (*), and the GFP is indicated by an arrow. (b) The GFP content of six independent samples (50 µg TSP) for each promoter was quantified by fluorimetry (excitation at 395 nm and emission at 508 nm). (PDF) [file pone.0115944.s002.pdf]

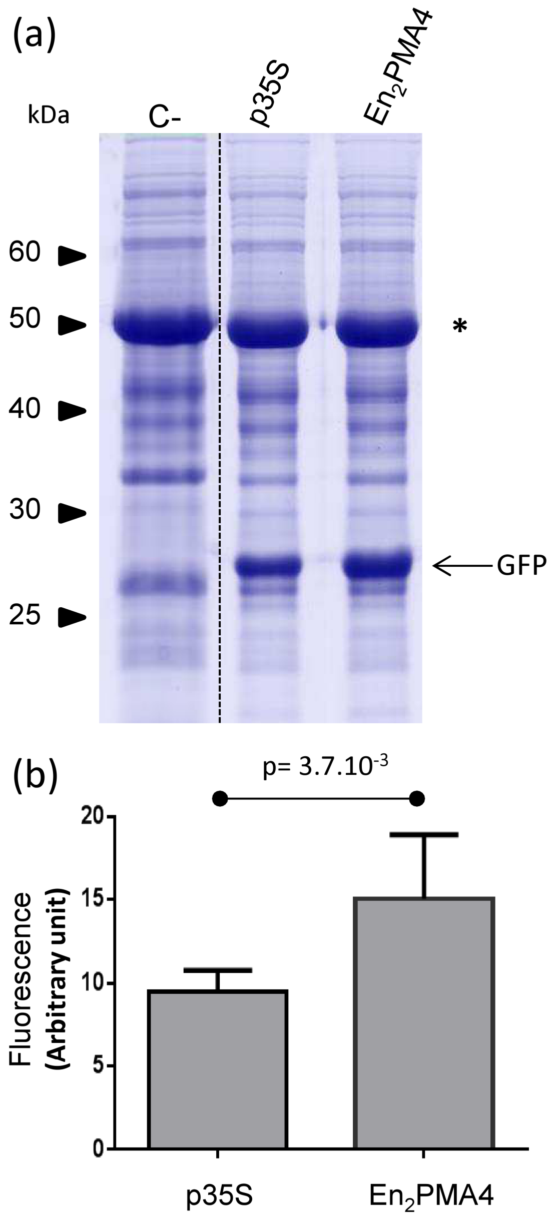


**Figure S2. Transient GFP expression comparison in *N. benthamiana* leaves between two strong transcriptional promoters.**

Leaves were infiltrated with an *A. tumefaciens* strain containing the pEAQ-HT vector with the *GFP* gene driven by the p35S or $En_2PMA4$ promoter. A leaf transformed with an empty pEAQ-HT vector was used as a negative control. A TSP fraction was prepared at 6 dpi. (a) Twenty µg of TSP were analyzed by SDS-PAGE and the gel was stained with colloidal blue. The large Rubisco subunit is indicated (*), and the GFP is indicated by an arrow. (b) The GFP content of six independent samples (50 µg TSP) for each promoter was quantified by fluorimetry (excitation at 395 nm and emission at 508 nm).
