# Supplementary material for: Hydrophobin Fusion of an Influenza Virus Hemagglutinin Allows High Transient Expression in Nicotiana benthamiana, Easy Purification and Immune Response with Neutralizing Activity
Source: PLoS One. 2014 Dec 26;9(12):e115944. doi: 10.1371/journal.pone.0115944 (PMC4277400; doi:10.1371/journal.pone.0115944)
Supplement: S3 Fig — Impact of acetosyringone in the infiltration medium on HA-HFBI accumulation. Following agroinfiltration with the H1-HFBI construct in the presence (+) or absence (−) of acetosyringone in the infiltration medium, total soluble proteins (TSP) were extracted at 6 dpi and the band corresponding to H1-HFBI was quantified by Western blotting. (n = 16, bars = SD, p-value = 0.0011). (PDF) [file pone.0115944.s003.pdf]

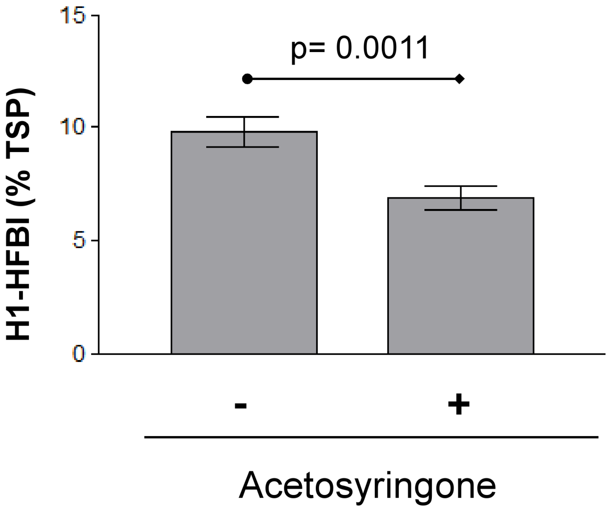

Figure S3. Impact of acetosyringone in the infiltration medium on HA-HFBI accumulation.

Following agroinfiltration with the H1-HFBI construct in the presence (+) or absence (-) of acetosyringone in the infiltration medium, total soluble proteins (TSP) were extracted at 6 dpi and the band corresponding to H1-HFBI was quantified by Western blotting. (n=16, bars = SD, p-value = 0.0011).
