# Supplementary material for: Hydrophobin Fusion of an Influenza Virus Hemagglutinin Allows High Transient Expression in Nicotiana benthamiana, Easy Purification and Immune Response with Neutralizing Activity
Source: PLoS One. 2014 Dec 26;9(12):e115944. doi: 10.1371/journal.pone.0115944 (PMC4277400; doi:10.1371/journal.pone.0115944)
Supplement: S4 Fig — Relative quantification of H1-HFBI and H1 accumulation. The H1 and H1-HFBI constructs were transiently expressed in N. benthamiana leaves. One construct was infiltrated on one half of the leaf and the other one on the other half. The H1-HFBI TSP fractions (1 µg) were diluted two, three, or four times and compared to undiluted H1 TSP fractions (1 µg) by Western blot analysis. Values obtained after quantification with the Kodak Image Station 4000R are displayed below each sample in arbitrary units. (DOCX) [file pone.0115944.s004.docx]

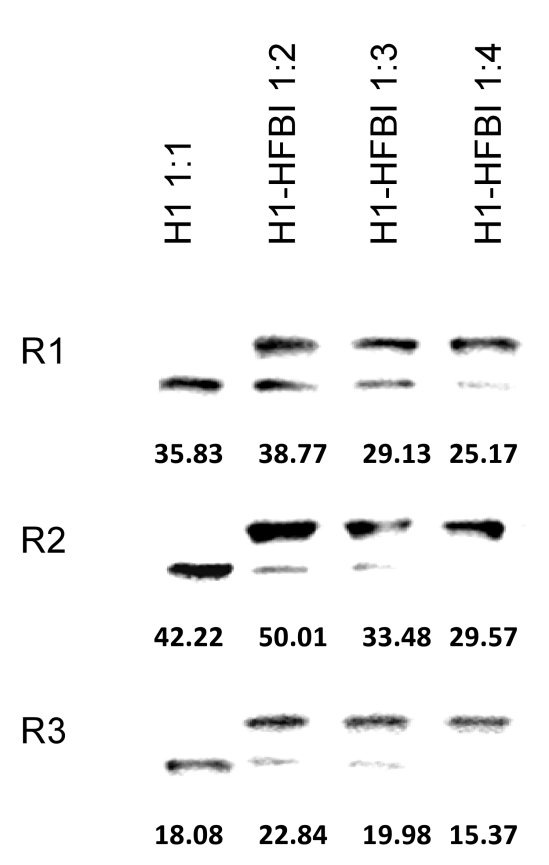


**Figure S4. Relative quantification of H1-HFBI and H1 accumulation**

The H1 and H1-HFBI constructs were transiently expressed in *N. benthamiana* leaves. One construct was infiltrated on one half of the leaf and the other one on the other half. The H1-HFBI TSP fractions (1 µg) were diluted two, three, or four times and compared to undiluted H1 TSP fractions (1 µg) by Western blot analysis. Values obtained after quantification with the Kodak Image Station 4000R are displayed below each sample in arbitrary units.
