# Supplementary material for: Hydrophobin Fusion of an Influenza Virus Hemagglutinin Allows High Transient Expression in Nicotiana benthamiana, Easy Purification and Immune Response with Neutralizing Activity
Source: PLoS One. 2014 Dec 26;9(12):e115944. doi: 10.1371/journal.pone.0115944 (PMC4277400; doi:10.1371/journal.pone.0115944)
Supplement: S5 Fig — Hemagglutination assay of purified HA-HFBI. Hemagglutination assay was performed as indicated in the Experimental procedures using serial two-fold diluted samples of dissolved ammonium sulfate precipitate of H1-HFBI (duplicate R1, R2). The upper row contains BSA as a negative control. (PDF) [file pone.0115944.s005.pdf]

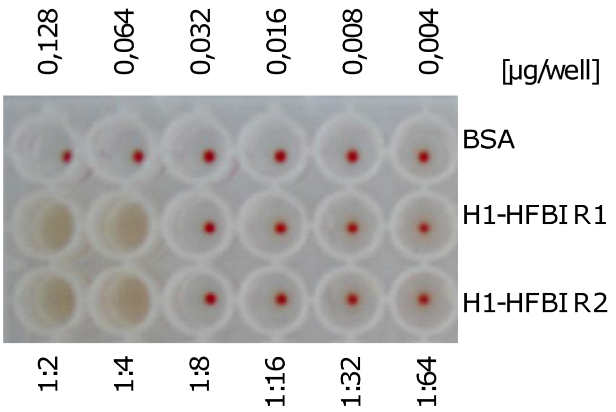

Figure S5: Hemagglutination assay of purified HA-HFBI.

Hemagglutination assay was performed as indicated in the Experimental procedures using serial two-fold diluted samples of dissolved ammonium sulfate precipitate of H1-HFBI (duplicate R1, R2). The upper row contains BSA as a negative control.
