# Supplementary material for: Hydrophobin Fusion of an Influenza Virus Hemagglutinin Allows High Transient Expression in Nicotiana benthamiana, Easy Purification and Immune Response with Neutralizing Activity
Source: PLoS One. 2014 Dec 26;9(12):e115944. doi: 10.1371/journal.pone.0115944 (PMC4277400; doi:10.1371/journal.pone.0115944)
Supplement: S6 Fig — Evaluation of anti-HFBI antibodies in immunized mouse serum. Samples containing H1-HFBI, GFP-HFBI or GFP (15 µg TSP) transiently expressed in N. benthamiana were analyzed by Western blotting using a 1∶200 diluted serum from mouse 6 immunized with H1-HFBI and a 1∶5000 dilution of a HRP-conjugated anti-mouse secondary antibody. The membrane was then stripped in 0.4N NaOH for 3 min and then incubated with a polyclonal anti-GFP antibody and a polyclonal anti-rabbit secondary antibody. Samples coming from a leaf infiltrated with an empty vector and a commercial recombinant H1 expressed in mammalian cells (rHA(+)) were used as negative and positive controls, respectively. Note thatadditional bands were detected with the mouse serum at a size similar to unfused GFP. They probably do not correspond to GFP as the fused GFP-HFBI is not recognized. (DOCX) [file pone.0115944.s006.docx]

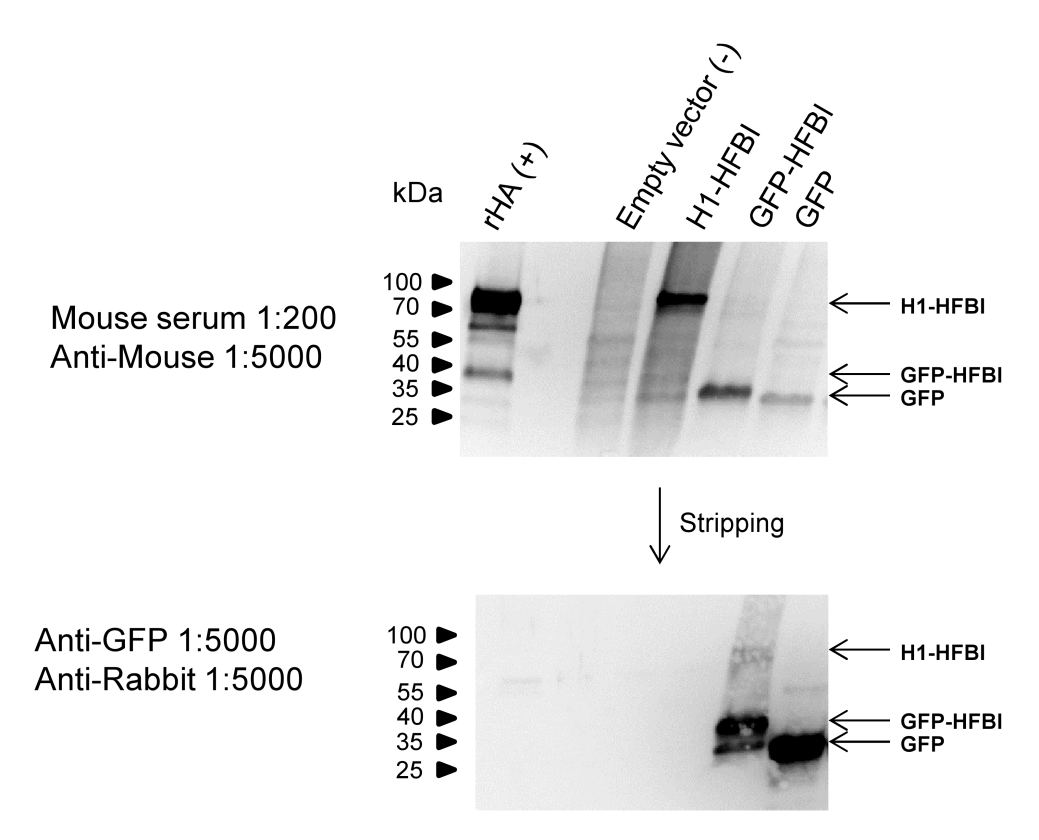


**Figure S6: Evaluation of anti-HFBI antibodies in immunized mouse serum.**

Samples containing H1-HFBI, GFP-HFBI or GFP (15 µg TSP) transiently expressed in *N. benthamiana* were analyzed by Western blotting using a 1:200 diluted serum from mouse 6 immunized with H1-HFBI and a 1:5000 dilution of a HRP-conjugated anti-mouse secondary antibody. The membrane was then stripped in 0.4N NaOH for 3 min and then incubated with a polyclonal anti-GFP antibody and a polyclonal anti-rabbit secondary antibody. Samples coming from a leaf infiltrated with an empty vector and a commercial recombinant H1 expressed in mammalian cells (rHA(+)) were used as negative and positive controls, respectively. Note thatadditional bands were detected with the mouse serum at a size similar to unfused GFP. They probably do not correspond to GFP as the fused GFP-HFBI is not recognized.
