# Supplementary material for: Hydrophobin Fusion of an Influenza Virus Hemagglutinin Allows High Transient Expression in Nicotiana benthamiana, Easy Purification and Immune Response with Neutralizing Activity
Source: PLoS One. 2014 Dec 26;9(12):e115944. doi: 10.1371/journal.pone.0115944 (PMC4277400; doi:10.1371/journal.pone.0115944)
Supplement: S1 Table — Identification of the proteins contaminating the ATPS-purified sample1. 1Method: The acquired spectra were analyzed using the Applied Biosystems GPS Explorer (version 3.6) and the Matrix Science MASCOT algorithm in the NCBI N. benthamiana database and the NCBI N. benthamiana EST database, as described in Duby et al. (2010). Reverse phase separation of peptides was completed on an Ultimate 3000 chromatography chain (ThermoFisher Scientific) using a C18 PepMap 100 analytical column (150 mm, 3 µm i.d., 100 Å), (ThermoFisher Scientific). Previously the sample was dissolved in 0.025% (v/v) TFA and 5% (v/v) ACN and desalted using a C18 Pep Map 100 pre-column (10 mm, 5 µm i.d., 100 Å). Peptides were backflushed onto the analytical column with a flow rate of 300 nL/min by a 180 min linear gradient from 8 to 76% (v/v) ACN in water containing 0.1% (v/v) TFA in buffer A and 0.085% (v/v) TFA in buffer B. The eluted peptides were mixed with a-cyano-4-hydrocinnamic acid (4 mg/mL in 70% ACN/0.1% TFA) and spotted directly onto a MALDI target using a Probot system (ThermoFisher Scientific). 2The band numbers correspond to those annotated in Fig. 5. (PDF) [file pone.0115944.s007.pdf]

Table S1. Identification of the proteins contaminating the ATPS-purified sample[1].

Band [2]	Accession number	Protein name	Size (kDa)
1	gi|384038819	chloroplast PsbO4 precursor	35.4
2	gi|61697113	chloroplast photosynthetic oxygen-evolving protein 23 kDa subunit	28.3
3	gi|511774224	2-Cys peroxiredoxin	25.5
4	gi|58700507	chloroplast oxygen-evolving protein 16 kDa subunit	24.2
5	gi|343174748	pathogenesis-related protein 1	17.7

[1]Method: The acquired spectra were analyzed using the Applied Biosystems GPS Explorer (version 3.6) and the Matrix Science MASCOT algorithm in the NCBI *N. benthamiana* database and the NCBI *N. benthamiana* EST database, as described in Duby et al. (2010).

Reverse phase separation of peptides was completed on an Ultimate 3000 chromatography chain (ThermoFisher Scientific) using a C18 PepMap 100 analytical column (150 mm, 3 µm i.d., 100 Å), (ThermoFisher Scientific). Previously the sample was dissolved in 0.025% (v/v) TFA and 5% (v/v) ACN and desalted using a C18 Pep Map 100 pre-column (10 mm, 5 µm i.d., 100 Å). Peptides were backflushed onto the analytical column with a flow rate of 300 nL/min by a 180 min linear gradient from 8 to 76% (v/v) ACN in water containing 0.1% (v/v) TFA in buffer A and 0.085% (v/v) TFA in buffer B.

The eluted peptides were mixed with a-cyano-4-hydrocinnamic acid (4 mg/mL in 70% ACN/0.1% TFA) and spotted directly onto a MALDI target using a Probot system (ThermoFisher Scientific).

[2]The band numbers correspond to those annotated in Figure 5.
